# Development of a Polymeric Film Entrapping Rose Bengal and Iodide Anion for the Light-Induced Generation and Release of Bactericidal Hydrogen Peroxide

**DOI:** 10.3390/ijms231710162

**Published:** 2022-09-05

**Authors:** Ana M. López-Fernández, Evelina E. Moisescu, Rosa de Llanos, Francisco Galindo

**Affiliations:** 1 Departamento de Química Inorgánica y Orgánica, Universitat Jaume I, Av. V. Sos Baynat s/n, 12071 Castellón, Spain; 2 Unidad Predepartamental de Medicina, Universitat Jaume I, Av. V. Sos Baynat s/n, 12071 Castellón, Spain

**Keywords:** photodynamic inactivation, singlet oxygen, hydrogen peroxide, iodide anion, triiodide anion, *E. coli*, *P. aeruginosa*, antibacterial materials, bactericidal polymers

## Abstract

A series of poly(2-hydroxyethyl methacrylate) (PHEMA) thin films entrapping photosensitizer Rose Bengal (RB) and tetrabutylammonium iodide (TBAI) have been synthetized. The materials have been characterized by means of Thermogravimetric Analysis (TGA), Attenuated Total Reflectance Fourier Transform Infrared Spectroscopy (ATR-FTIR) and UV-vis Absorption spectroscopy. Irradiation of the materials with white light led to the generation of several bactericidal species, including singlet oxygen (^1^O_2_), triiodide anion (I_3_^−^) and hydrogen peroxide (H_2_O_2_). ^1^O_2_ production was demonstrated spectroscopically by reaction with the chemical trap 2,2′-(anthracene-9,10-diylbis(methylene))dimalonic acid (ABDA). In addition, the reaction of iodide anion with ^1^O_2_ yielded I_3_^−^ inside the polymeric matrix. This reaction is accompanied by the formation of H_2_O_2_, which diffuses out the polymeric matrix. Generation of both I_3_^−^ and H_2_O_2_ was demonstrated spectroscopically (directly in the case of triiodide by the absorption at 360 nm and indirectly for H_2_O_2_ using the xylenol orange test). A series of photodynamic inactivation assays were conducted with the synthesized polymers against Gram-negative bacteria *Escherichia coli* and *Pseudomonas aeruginosa*. Complete eradication (7 log_10_ CFU/mL) of both bacteria occurred after only 5 min of white light irradiation (400–700 nm; total energy dose 24 J/cm^2^) of the polymer containing both RB and TBAI. The control polymer without embedded iodide (only RB) showed only marginal reductions of ca. 0.5 log_10_ CFU/mL. The main novelty of the present investigation is the generation of three bactericidal species (^1^O_2_, I_3_^−^ and H_2_O_2_) at the same time using a single polymeric material containing all the elements needed to produce such a bactericidal cocktail, although the most relevant antimicrobial activity is shown by H_2_O_2_. This experimental approach avoids multistep protocols involving a final step of addition of I^−^, as described previously for other assays in solution.

## 1. Introduction

Infections caused by antibiotic-resistant microorganisms constitute a global threat to modern health systems and economies [1,2]. Diverse chemical strategies are under investigation to stop the spreading of such pathogens [3,4]. Antimicrobial photodynamic inactivation (aPDI) has emerged as a promising tool to combat the threat of these microbes. aPDI relies on the use of an appropriate photosensitizer molecule capable of activation by light, and generation of reactive oxygen species (ROS) via the two well-known type I (electron-transfer) and type II (energy-transfer) mechanisms [5,6,7,8,9,10,11,12,13,14]. The generated ROS can destroy the pathogenic cells in different ways such as attacking the cellular envelope (major pathway) or damaging proteins and nucleic acids (to a minor extent) [15,16,17,18]. ROS include superoxide anion (O_2_^−^), hydroxyl radical (HO^·^), hydrogen peroxide (H_2_O_2_) and singlet oxygen (^1^O_2_) [19,20]. It must be noted that aPDI principles also apply in the field of Photodynamic Therapy (PDT) of cancer [21,22].

The field of aPDI is growing rapidly, and new photosensitizing molecules operating in aqueous solution are reported every year [23,24]. In parallel, solid materials, especially polymers with grafted or embedded photosensitizers, have been described abundantly [4,25,26,27,28,29,30]. These materials are expected to become the future components of real devices, from self-sterilizing catheters or prothesis to cell phone screen protectors, coatings for doorknobs or wall paints for hospitals, to mention only a few envisaged examples. In this regard, we have reported in the past some polymeric photoactive systems able to kill planktonic cultures of *Staphylococcus aureus* [31,32,33], *Pseudomonas aeruginosa* [32,33,34,35], *Escherichia coli* [33] and *Enterococcus faecalis* [33], and biofilms of *S. aureus* [36].

The discovery that the generation of cytotoxic species against microorganisms can be boosted by the addition of some inorganic salts has revolutionized the field of aPDI recently [37,38,39]. This is especially true for iodide, an anion that is harmless from the viewpoint of human health. The use of iodide in high concentration (typically 100 mM) improved dramatically the killing of certain microbial species [39,40,41,42,43,44,45,46,47,48,49,50,51,52,53,54,55,56,57,58]. However, the typical operational procedure in most of the reported papers consists of the addition of an iodide salt, normally potassium iodide (KI), which is dissolved in the bacterial growth medium, with subsequent light irradiation to produce ^1^O_2_ and species derived from the reaction of ^1^O_2_ with I^−^, like peroxyiodide (HOOI_2_^−^), iodide radical (I_2_^−^), iodine (I_2_), triiodide (I_3_^−^), hydrogen peroxide (H_2_O_2_), hydroperoxide radical (HOO), etc. In other words, both the soluble photosensitizer and the adjuvant iodide are present in the solution at the moment of irradiation. Taking into account that the rate of reaction between ^1^O_2_ and I^−^ is rather slow (9.2 × 10 ^5^ M^−1^ s^−1^) [44], it is not surprising that high concentrations of KI (100 mM) are needed to observe antimicrobial activity. To solve this shortcoming, recently, a clever strategy bringing photosensitizer and I^−^ in close proximity by encapsulation of both inside lipidic micelles has been reported [52]. By means of this approach the concentration of both photosensitizer and I^−^ needed to achieve pathogen killing (*Candida albicans*) was notably reduced. Later, this formulation was applied successfully in the area of PDT to eliminate melanoma cancerous cells [57]. However, lipidic micelles are not usable in most of the real-life materials expected to find application in the medical context (a catheter, for instance) or quotidian objects (a mobile phone case, for instance). In this paper, we describe the incorporation of both the ^1^O_2_ photosensitizer Rose Bengal (RB) and the iodide salt tetrabutylammonium iodide (TBAI) into a polymeric matrix of crosslinked poly(2-hydroxyethyl methacrylate) (PHEMA). White light irradiation of this material generates at least three cytotoxic species: ^1^O_2_, I_3_^−^ and H_2_O_2_, and the last one is released from the polymer, conferring a long-lasting bactericidal effect after the light is switched off. To the best of our knowledge, no polymeric materials have been reported so far generating this cocktail of biocidal species at the same time. The combination here described has proved to be very successful for the complete eradication (7 log CFU/mL) of Gram negative *E. coli* and *P. aeruginosa* bacteria.

## 2. Results and Discussion

### 2.1. Synthesis and Characterization of PHEMA Films

**Figure 1:**
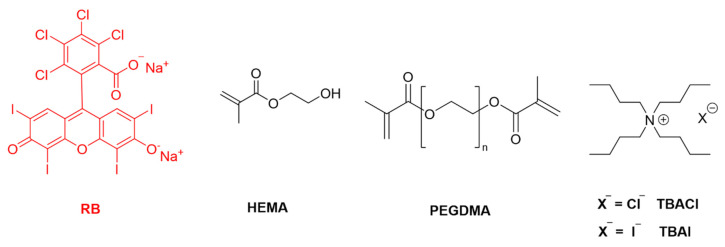
Structures of Monomer (HEMA), Crosslinker (PEGDMA), Photosensitizer (RB) and Tetrabutylammonium Halide Salts Employed to Synthesize the PHEMA Films Described in This Work.

Synthesis of PHEMA films was carried out following the methodology described previously to prepare similar polymers [35,36,59,60,61,62,63], some of them used in photodynamic studies [35,36]. Briefly, a mixture of 2-hydroxyethyl methacrylate monomer (HEMA, 850 weight parts) and crosslinker poly(ethylene glycol) dimethacrylate (PEGDMA, 150 weight parts) was prepared. To this homogeneous mixture, RB and initiator AIBN were added (10 weight parts) and stirred till dissolution. Then TBA salt (chloride or iodide) was added and stirred again. For one case sodium iodide was added, instead of TBAI, to have a polymer containing an inorganic salt to compare with TBAI. Formulas of HEMA, PEGDMA, RB and TBA halides are shown in Figure 1. The proportion of each component of the polymerizable mixture can be seen in Table 1.

**Figure 2:**
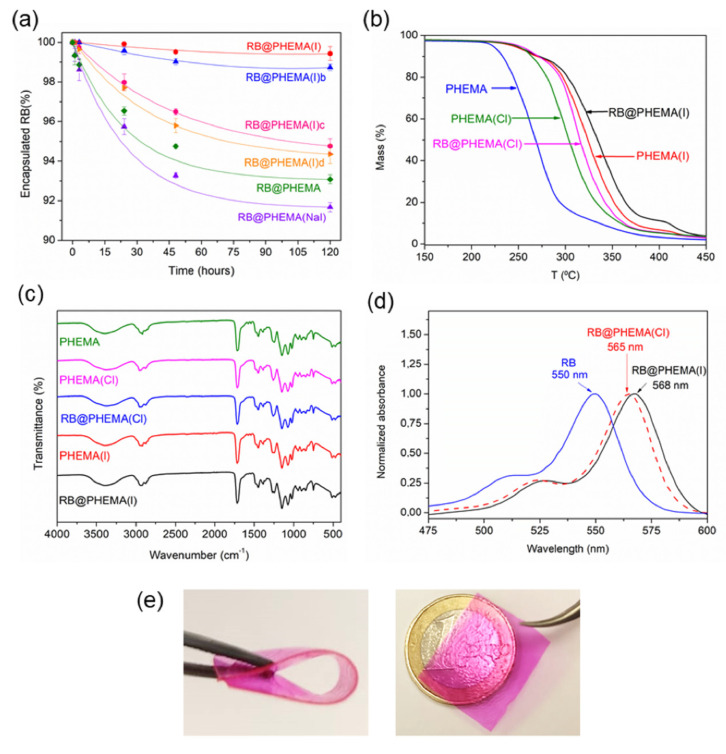
Characterization of PHEMA Films Described in this Study: (**a**) leaching of RB; (**b**) TGA curves; (**c**) ATR-FTIR spectra; (**d**) UV-vis absorption and (**e**) representative pictures of polymer RB@PHEMA(I).

Polymerization carried out made inside molds fabricated with two glass slides and two coverslips (thickness ca. 120 μm), leading to films of dimensions ca. 75 mm × 25 mm (later cut into smaller pieces of 25 mg or 50 mg each). The prepared films were all transparent and flexible but different in their ability to retain RB when soaked in aqueous solutions. As can be seen in Figure 2a, polymers containing RB without additive (RB@PHEMA), and polymers made with NaI as an additive (RB@PHEMA(NaI)) lost about 7–8% of dye in about 2 h. However, polymers made with TBAI (RB@PHEMA(I), RB@PHEMA(I)b, RB@PHEMA(I)c, RB@PHEMA(I)d) retained better the photosensitizer, with RB@PHEMA(I), made with ca. 10% TBAI, showing a minimal loss of only 1% after 2 h. Hence, washing these materials after polymerization releases the untrapped RB and is ready for use. A notable difference with this material, as compared to previously reported films [35], is that in the past the cationic monomer (3-acryalamidopropyl)trimethyl ammonium chloride (ATAC) was used in a small proportion (5 wt.%), in order to provide a cationic anchoring point for anionic photosensitizer RB. For the polymers now reported it was decided not to use that monomer in order to keep the monomer/crosslinker formulation as simple as possible (only HEMA and PEGDMA). It was hypothesized that tetrabutylammonium cation, trapped within the entangled chains of PHEMA, would act as such an anchoring point for RB as well. According to the excellent stability to leaching showed by RB@PHEMA(I) it seems clear that the function made by ATAC in previous films to retain RB anion is made by TBA cation in the present case to retain both RB and I^−^ anions.

The addition of TBA salts can also be noticed in the recorded thermal stability of the films. Thermogravimetric (TGA) data of polymers PHEMA, RB@PHEMA(NaI), RB@PHEMA(Cl) and RB@PHEMA(I) are shown in Figure 2b and in Table 2. The addition of RB and TBAI has a notable reinforcement effect, relative to the basic PHEMA matrix, with an increase of about 50 °C in the decomposition temperature. This fact supports the idea that TBA cations remain firmly entrapped within the PHEMA matrix, acting as some type of non-covalent crosslinker.

Attenuated Total Reflectance Fourier Transform Infrared Spectroscopy (ATR-FTIR) was measured for the five polymers above mentioned and the expected bands were recorded: hydroxyl stretching around 3700–3100 cm^−1^, carbonyl stretching around 1712 cm^−1^ and C-O vibrations in the range 1300–1000 cm^−1^ (see Figure 2c). Regarding UV-vis spectroscopic characterization, the high transparency of the synthesized films allows easy measurement of the absorption bands of the entrapped RB. As can be seen in Figure 2d, RB peaked at 568 nm inside film RB@PHEMA(I) and at 565 nm inside film RB@PHEMA(Cl). Those bands are redshifted compared to RB in free aqueous solution (550 nm), which is a clear indication of the location of this photosensitizer in a more hydrophobic environment [35]. In addition, it must be said that the shape of the spectra of RB inside the films is not particularly broadened due to aggregation phenomena [64], which would interfere with the photogeneration of ^1^O_2_. Pictures of a film of RB@PHEMA(I) are shown in Figure 2e in order to illustrate the flexibility and transparency of the materials under study.

### 2.2. Photochemical Generation of Singlet Oxygen, Triiodide Anion and Hydrogen Peroxide by PHEMA Films

**Figure 3:**
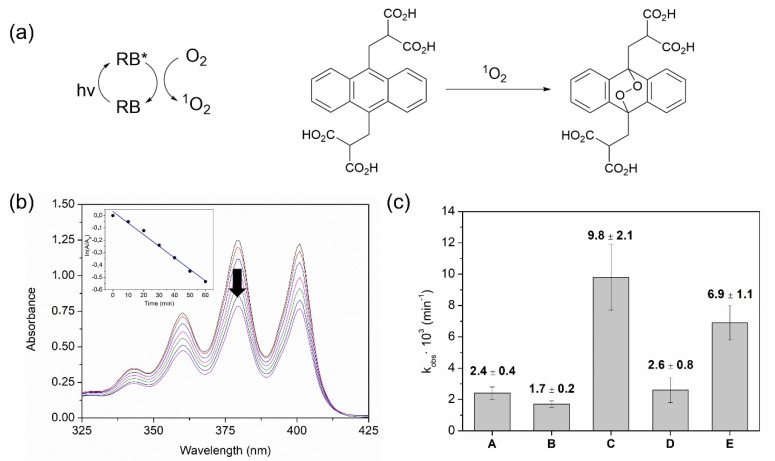
Generation of ^1^O_2_ by Irradiated PHEMA Films: (**a**) photosensitization cycle involving excitation of RB/energy transfer to oxygen and reaction of generated ^1^O_2_ with ABDA as chemical trap (arrow indicates the evolution of the absorption with time); (**b**) Representative change of the UV-vis absorption spectra of ABDA upon reaction with ^1^O_2_; (**c**) Calculated kinetic constants of the reaction between ABDA and ^1^O_2_ generated by different PHEMA films, where A: PHEMA; B: PHEMA(I), C: RB@PHEMA(I); D: PHEMA(Cl) and E: RB@PHEMA(Cl).

Films with entrapped RB (with or without TBA salts) were submitted to irradiation with white light (400–700 nm) to evaluate their ability to generate ^1^O_2_. To test this property, the water-soluble chemical trap of ^1^O_2_ 2,2′-(anthracene-9,10-diylbis(methylene)) dimalonic acid (ABDA) was employed. The UV-vis absorption of this probe fades out upon reaction with ^1^O_2_ due to the formation of an endoperoxide, and hence the evolution of the reaction can be easily monitored [65]. The oxygenation reaction and the photosensitization cycle are depicted in Figure 3a. This process follows pseudo-first-order kinetics, described by the expression ln (A/A_0_) = −k_obs_ t, where A_0_ is the initial absorbance of ABDA, A is the absorbance of ABDA at time t and k_obs_ is the observed kinetic constant. Thus, the determination of k_obs_ for each polymer allows an estimation of the capabilities of each film to generate ^1^O_2_. RB-containing polymers showed values of this constant in the range 6.9–9.8 × 10^−3^ min^−1^, whereas control polymers without RB (PHEMA, PHEMA(Cl), PHEMA(I)) performed the oxygenation of ABDA with k_obs_ = 1.7–2.6 × 10^−3^ min^−1^ (attributable to self-sensitized oxygenation of ABDA). In comparison to previously reported systems [31,32,35], these numbers seem apparently low but it must be recalled that for this study the reactivity has been measured directly in water, where the lifetime of ^1^O_2_ is notably lower than in organic solvent (in previous works 9,10-dimethylanthracene was used as a probe in acetonitrile). Figure 3b shows a representative example of this methodology and in Figure 3c the measured k_obs_ for all the irradiations are depicted. Nevertheless, the use of soluble probes to test the ability of polymers to generate ^1^O_2_ must be taken with caution, since the generation of this ROS occurs inside the matrix and at the surface of the polymer, and owing to the short lifetime of ^1^O_2_, the distance that it can travel is very short (a few microns). Additionally, the novelty of the material RB@PHEMA(I) here described consists of the addition of TBAI as a part of the formulation, which implies that most of the photogenerated ^1^O_2_ could react with I^−^ within the polymer and hence likely not all the ^1^O_2_ produced could be detected, but the remaining after reaction with I^−^.

As indicated before, the novelty of the materials now reported resides in the potential formation of a collection of bactericidal species, namely ^1^O_2_ (obtained by photosensitization), I_3_^−^ and H_2_O_2_ (both obtained indirectly) inside or at the surface of the matrix. The set of reactions leading to extra bactericidal species starts with the interaction of ^1^O_2_ with I^−^, as can be seen in Equations (1)–(5). Further details can be obtained from the pertinent literature describing assays combining photosensitizers with iodide in aPDI studies [40,41,42,43,44,45,46,47,48,49,50,51,52,53,54,55,56,57,58].

^1^O_2_ + I^−^ + H_2_O → IOOH + HO^−^ (1)

IOOH + I^−^ → HOOI_2_^−^ (2)

HOOI_2_^−^ → I_2_ + HO_2_^−^ (3)

I_2_ + I^−^ → I_3_^−^ (4)

HO_2_^−^ + H_2_O → H_2_O_2_ + HO^−^ (5)

**Figure 4:**
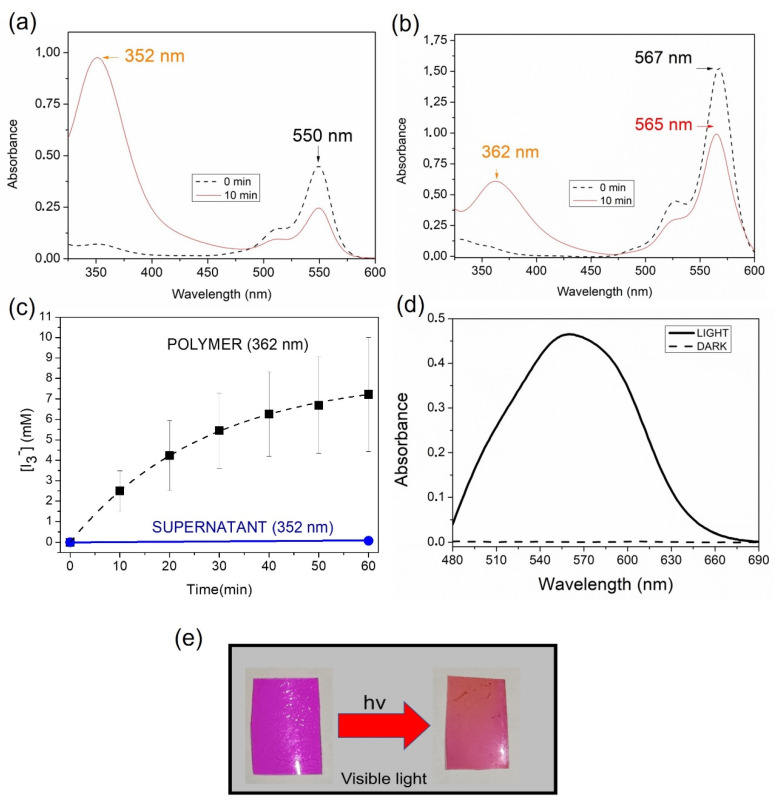
Generation of I_3_^−^ Anion upon Irradiation of (**a**) RB Aqueous Solution (5 µM) Plus KI (100 mM) and (**b**) Film RB@PHEMA(I). (**c**) Evolution of Concentration of I_3_^−^ in Film RB@PHEMA(I) and Supernatant. (**d**) Detection of H_2_O_2_ in the Supernatant by Means of Xylenol Orange Method (See Experimental Section; Case of RB@PHEMA(I) Irradiated for 1 h). (**e**) Pictures of Films of RB@PHEMA(I) Before (**left**) and After (**right**) Irradiation.

The generation of I_3_^−^ after illumination of RB@PHEMA(I) was proved by recording the UV-vis absorption spectra of the film after irradiation. As it can be seen in Figure 4a, after only 10 min of irradiation, an intense band at 362 nm appeared, typical of I_3_^−^ species [66]. For comparison purposes, the same experiment was made using RB (5 µM) and potassium iodide (100 mM) in solution. As can be seen, a peak raised intensely after only 10 min of irradiation, but at 352 nm (Figure 4b). The difference in position of the maxima with respect RB@PHEMA(I) must be attributed to the different microenvironments in which the anion is located (aqueous vs. polymeric) [35]. It is worth noting that the generated I_3_^−^ remained entrapped in the polymer after 1 h of irradiation and the appearance of the films before and after irradiation allows one to appreciate visually the change (darker films after irradiation; see Figure 4e). An interesting observation is the absence of a band at 460 nm, which is characteristic of iodine (I_2_) [66]. Importantly, no I_3_^−^ in the supernatant solution (352 nm) was detected, indicative of complete retention of this anion inside the matrix, presumably paired with TBA cation. Considering the molar extinction coefficient of I_3_^−^ in water (2.2 × 10^4^ M^−1^ cm^−1^) [67] and the thickness of the films, allows recording the evolution of the concentration of I_3_^−^ inside the material, reaching up to ca. 8 mM after 1 h of irradiation (Figure 4c). The presence of H_2_O_2_ in the supernatant was demonstrated by using the spectrophotometric method of xylenol orange in acidic medium (see Figure 4d for the case of RB@PHEMA(I) irradiated for 1 h) [68,69].

### 2.3. Photodynamic Activity of PHEMA Films against P. aeruginosa and E. coli

**Figure 5:**
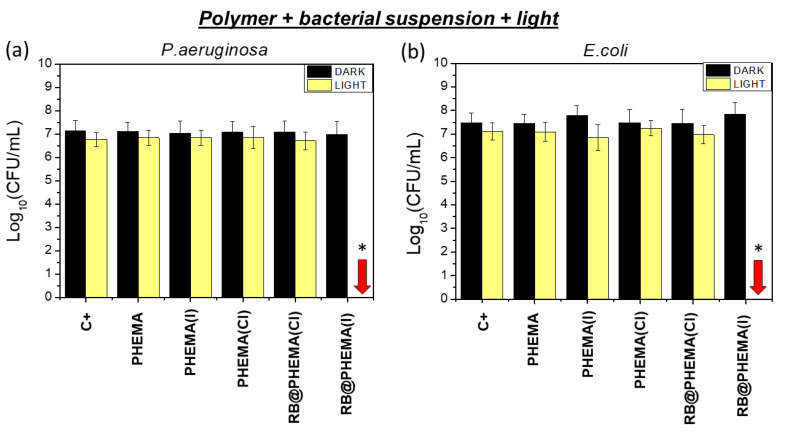
Photodynamic Inactivation Assays (5 min irradiation, 24 J/cm^2^) against *P. aeruginosa* ATCC 27853 (**a**) and *E. coli* CECT 101 (**b**) Using Polymer PHEMA Materials. Bacterial cell survival was evaluated by counting colony-forming units (CFU) on MHA at 35 °C for 24 h. The results are expressed as mean ± standard deviation. Asterisk (*) indicates significant differences (*p* < 0.01). Red arrow indicates complete eradication.

Planktonic suspensions (ca. 7 log CFU/mL initial load) of Gram-negative *P. aeruginosa* and *E. coli* bacteria were exposed to irradiation with white light (400–700 nm, 80 mW/cm^2^) in the presence of polymers with RB and TBA salts (RB@PHEMA(I) and RB@PHEMA(Cl)). Also control polymers without photosensitizer (PHEMA, PHEMA(I) and PHEMA(Cl)) were used. Irradiation for 5 min (24 J/cm^2^ total energy dose) resulted in significant complete eradication (*p* < 0.01) of both bacteria populations, only when RB@PHEMA(I) was used (Figure 5). In the case of irradiated RB@PHEMA(Cl) and PHEMA(I), PHEMA(Cl) or PHEMA, only marginal and non-significant decreases in bacterial population were detected (less than 0.5 log CFU/mL). Control experiments in the dark resulted in no bactericidal effect, irrespective of the conditions used (PHEMA, PHEMA(Cl), PHEMA (I), RB@PHEMA(Cl) or RB@PHEMA(I)).

**Figure 6:**
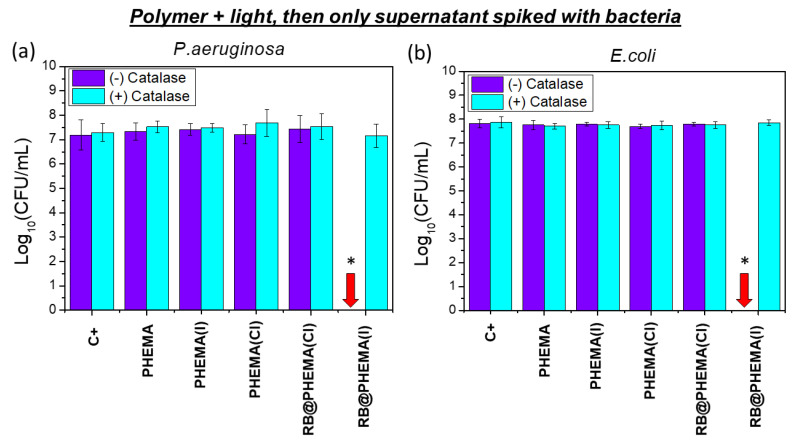
Bactericidal Ability of the Supernatants against *P. aeruginosa* ATCC 27853 (**a**) and *E. coli* CECT 101 (**b**). Polymers submerged in water were first irradiated for 1 h (288 J/cm^2^ of total energy dose) and then removed from the solution. The remaining supernatants were split into two aliquots. One aliquot was treated with catalase ((+) catalase) and the other not ((-) catalase), and then allowed to react for 1 h. Then, each aliquot was spiked with a bacterial suspension of either *P. aeruginosa* ATCC 27853 and *E. coli* CECT 101 and incubated for 5 min. Bacterial cell survival was evaluated by counting colony-forming units (CFU) on MHA at 35 °C for 24 h. The results are expressed as mean ± standard deviation. Asterisk (*) indicates significant differences (*p* < 0.01). Red arrow indicates complete eradication.

Three main conclusions emerge from these assays:

(a) Polymers PHEMA(Cl) and PHEMA(I) are not able to induce bacterial mortality, which implies that, despite quaternary ammonium cations (QACs) are paradigmatic examples of species with well-recognized biocidal properties [70], in our case PHEMA entrapping TBA cation do not display such property.

(b) RB alone inside PHEMA (without iodide, i.e., polymer RB@PHEMA(Cl)) cannot generate sufficient cytotoxic species, when irradiated, to induce appreciable bacterial death at 5 min. of irradiation time. This would mean that the amount of generated ^1^O_2_ is not enough to cause a noticeable bactericidal effect in this short irradiation time. However, it must be noted that prolonged irradiation times (1 h) did cause appreciable reductions in bacterial populations. 

(c) The simultaneous presence of both RB and I^−^ inside the polymeric hydrogel (polymer RB@PHEMA(I)) induces a strong bactericidal effect, causing a complete eradication (7 log CFU/mL) of the bacterial populations of both Gram negative bacteria *E. coli* and *P. aeruginosa* (red arrows in Figure 5a,b). The cause of such notable killing effect could be ascribed, in principle, to any of the bactericidal species generated under such conditions, namely ^1^O_2_, I_3_^−^ and H_2_O_2_. However, as discussed above (point (b)), the amount of ^1^O_2_ generated seems to be insufficient to account for such observation. This renders I_3_^−^ and H_2_O_2_ as potentially responsible for the observed effect, however, I_3_^−^ remains entrapped in the polymeric matrix (see spectroscopic determinations described above), and hence only the trapped but superficial I_3_^−^ in close contact with the solution is expected to be bactericidal. It seems unlikely that the I_3_^−^ retained deep into the matrix would cause any bactericidal effect. Following this reasoning, the generated H_2_O_2_, which would be produced inside the polymer but could diffuse into the supernatant solution, might be responsible for the observed bacterial population reduction. In order to test this hypothesis, a series of irradiations for 1 h were performed with all the studied polymers immersed in water but without bacteria. Afterward, the polymers were removed and the supernatant solutions were spiked with an aliquot of either *E. coli* or *P. aeruginosa* suspensions (up to 7 log CFU/mL). As can be seen in Figure 6a,b, only the supernatant of irradiated RB@PHEMA(I) resulted as bactericidal (7 log CFU/mL reduction), which points out to H_2_O_2_ as the responsible, at least in part, of such effect. In another series of assays, catalase was used as quencher [71] of the generated H_2_O_2_ in the same conditions (Figure 6, assays labeled as (+) catalase), and, as expected, the observed bacterial reduction for both bacteria was negligible. It must be recalled, that H_2_O_2_ is a very well-known antiseptic and its use against Gram positive and negative bacteria has been deeply studied [72,73], however, at the present stage of the investigation it cannot be discarded that other biocidal species derived from H_2_O_2_ (since catalase is able to block the activity in the supernatant) are playing some role. It‘s worth to be considered that H_2_O_2_ and I^−^ can further react to yield IOOH, HOOI_2_^−^ and ultimately I_2_^−^ and HOO^·^ radicals (Equations (6)–(8)), as described by Dalmazio et al. [74], which could have some bactericidal contribution. However, detection of those species was not possible and hence their concentrations cannot be estimated or their role in the biological effect determined.

I^−^ + H_2_O_2_ + H^+^ → IOOH + 2H_2_O (6)

IOOH + I^−^ → HOOI_2_^−^ (7)

HOOI_2_^−^ → I_2_^−^ + HOO^·^ (8)

To sum up, neither ^1^O_2_ (which is too short-lived) nor I_3_^−^ (which remains entrapped in the matrix) could be directly responsible for the observed bactericidal effect shown in this study, but H_2_O_2_, diffused out of the hydrogel (and/or some downstream species derived from H_2_O_2_) might be crucial in the observed bactericidal result. This investigation, hence, highlights the importance of species different from ^1^O_2_ and I_3_^−^ as responsible for the notable killing effect described in the last years, when photosensitizers and I^−^ are combined in aPDI studies. The material here described could be added to the growing arsenal of polymers with H_2_O_2_-releasing properties for antimicrobial applications [75,76,77,78].

## 3. Conclusions

In summary, polymer RB@PHEMA(I) made with HEMA, PEGDMA, TBAI and RB has been synthesized and fully characterized by means of ATR-FTIR, TGA and UV-vis spectroscopy. Also, control materials without RB or I^−^ were also produced. The irradiation of RB@PHEMA(I) with white light generates ^1^O_2_, which, upon reaction with I^−^, triggers a series of reactions leading to I_3_^−^ and H_2_O_2_ (at least). ^1^O_2_ and H_2_O_2_ were detected indirectly by means of spectroscopic probes (ABDA and xylenol orange, respectively), whereas the absorption bands of I_3_^−^ were detected directly by UV-vis spectroscopy. The anion I_3_^−^ remains entrapped into the polymeric matrix and some of the H_2_O_2_ diffuses from the material to the supernatant solution. The aPDI assays against Gram negative bacteria *P. aeruginosa* and *E. coli* show that this polymer is able to eradicate bacterial populations of 7 log CFU/mL after only 5 min. of irradiation (white light, energy dose 24 J/cm^2^) and importantly, control polymers with only I^−^ or RB do not cause any bacterial killing. Hence, it can be assumed that the combination of photosensitizer and I^−^ inside the polymer is key to understanding the notable biocidal effect. The microbiological assays made with only the supernatants from irradiated polymers resulted positive only in the case of RB@PHEMA(I), which suggests that the key bactericidal species leaches out from the polymer and has a considerable long lifetime, then suggesting that it is H_2_O_2_ or some species derived from it (catalase addition halts the bacterial killing). We hope that the findings here reported would help to understand the increasing number of investigations in the realm of aPDI involving the joint use of photosensitizers and iodide. Also, it is expected that RB@PHEMA(I), or other materials inspired by it, will help to design and produce polymers with photo-antimicrobial properties applicable to the manufacture of real-life objects prone to be a source of contagion by contact.

## 4. Materials and Methods

### 4.1. Materials

Rose Bengal sodium salt (RB, MW: 1017.64 g/mol, ≥85%, Sigma, Madrid, Spain), tetrabutylammonium iodide (TBAI, MW: 369.37 g/mol, 98%, Aldrich Chemistry, Madrid, Spain), tetrabutylammonium chloride (TBACl, MW: 277.92 g/mol, ≥97%, Aldrich Chemistry, Madrid, Spain), Sodium iodide (NaI, Fisher Chemical, Madrid, Spain), 2-hydroxyethyl methacrylate (HEMA, MW: 130.14 g/mol, 97%, Acros Organics, Madrid, Spain), poly(ethylene glycol) dimethacrylate average Mn 550 (PEGDMA, Aldrich Chemistry, Madrid, Spain), 2,2′-azo-bis-isobutironitrile (AIBN, 98%, Merck, Madrid, Spain), 9,10-anthracenediyl-bis(methylene) dimalonic acid (ABDA, ≥90% (HPLC)), sodium phosphate monobasic dihydrate (≥99%, Sigma-Aldrich, Madrid, Spain), sodium phosphate dibasic anhydrous (≥99%, Fluka, Madrid, Spain), catalase from bovine liver (aqueous suspension, 10.000–40.000 units/mg protein, Sigma-Aldrich, Madrid, Spain), ammonium iron (II) sulfate hexahydrate (for analysis, Thermo Scientific, Madrid, Spain), xylenol orange sodium salt (Thermo Scientific, Madrid, Spain), sorbitol (≥97%, Fisher, Madrid, Spain), sulphuric acid (96%, Merck, Madrid, Spain) and hydrogen peroxide (for analysis, 35 wt.% solution in water, stabilized, Thermo Scientific, Madrid, Spain). All reagents were used as received.

### 4.2. Polymer Synthesis

The polymeric materials were made following the protocol previously described: ^35^RB sodium salt (1 mg) and AIBN (10 mg) were added to a mixture of HEMA (850 mg), PEGDMA (150 mg). Subsequently, TBACl or TBAI (from 10 to 100 mg in this case) was incorporated and dissolved. The resultant solution was inserted into a rectangular mold made with two microscope slides separated by two coverslips (120 μm thickness). Then, the mold was put in an oven at 85 °C for 15 min. The polymeric film was obtained when the two microscope slides were separated. The material was cleaned with distilled water to eliminate unreacted monomer and crosslinker. Prior to polymerization, the glass microscope slides were conditioned by treatment with silicone oil and butanone (1% wt. relative to silicone oil) and introduced in an oven at 200 °C for 60 min.

### 4.3. Characterization

The PHEMA films were characterized by Attenuated Total Reflectance Fourier Transform Infrared Spectroscopy (ATR-FTIR) with a Jasco FT/IR 6200 type A apparatus with a TGS detector. The range of the spectra ATR-FTIR was 4000–400 cm^−1^, with 128 scans per spectrum (4 cm^−1^ resolution). Thermogravimetric analyses (TGA) were carried out with a TG-STDA Mettler Toledo apparatus model TGA/SDTA851e/LF/1600 from 25 °C to 500 °C, at a heating rate of 10 °C/min under air atmosphere. All assays were performed in 40 µL platinum crucibles and an empty platinum crucible was employed as a reference. Sample masses of ca. 10 mg were used. The UV-vis absorption measurements of polymeric films were made on an Agilent Cary 60 UV-vis spectrophotometer.

### 4.4. Photochemical Studies

Photochemical oxidation reactions were carried out inside 10 mL vials containing 50 mg of polymeric film and 3 mL of ^1^O_2_ probe solution (ABDA 10^−4^ M in 1 mM phosphate buffer, pH 7.4). The system of irradiation consisted of two white light LED lamps (9 W each, 400–700 nm emission output, light irradiance 180 mW/cm^2^ for each lamp) positioned 2.5 cm away from the reaction vials and under constant stirring. The reaction of photooxygenation of ABDA was monitored by UV-Vis absorption spectroscopy at different times (from 0 to 60 min). The production of H_2_O_2_ was estimated by the xylenol orange spectrophotometric detection method [68,69,79].

### 4.5. Microorganisms and Growth Conditions

The Gram (-) bacterial strains *P. aeruginosa* ATCC 27853 and *E. coli* CECT 101 were supplied by the American Type Culture Collection (ATCC, Rockville, MD, USA) and The Colección Española de Cultivos Tipo (CECT, Valencia, España). Bacterial growth was carried out aerobically overnight at 35 °C in Mueller Hilton Agar (Scharlau, Spain).

### 4.6. Antimicrobial Photodynamic Inactivation Assays

The antimicrobial photodynamic inactivation (aPDI) was performed by exposing the bacterial suspension to light and the corresponding photoactive polymer (25 mg) (PHEMA, PHEMA(Cl), PHEMA (I), RB@PHEMA(Cl) or RB@PHEMA(I)). The light source for these assays was a LED TENKO ECO lamp (50 W, 400–700 nm, light irradiance 80 mW/cm^2^), placed 2.5 cm from the samples. All experiments included a positive growth control, in which cells were incubated without any of the aforementioned films.

These six groups (PHEMA, PHEMA(Cl), PHEMA(I), RB@PHEMA(Cl) or RB@PHEMA(I) and positive growth control) were subjected to irradiation. Moreover, in parallel, the same groups were kept in darkness as controls. Previously to the aPDI experiments, all films were first sterilized by dipping them in 70% ethanol and subsequently air-dried. Then, each polymer was located in a well of a 12-well sterile plate [35]. Bacterial inoculum for both bacteria species was prepared in sterilized distilled water and adjusted to 0.50 ± 0.03 on the McFarland scale (microbial suspensions containing approx. 1.5 × 10^8^ bacteria/mL). An aliquot of 1 mL of the bacterial suspension was equally distributed to the 12-well plates (for irradiation and for dark conditions). Irradiation of the 12-well plate containing films and controls were exposed 5 min to white light (24 J/cm^2^ of total light dose), at room temperature and under agitation in an orbital shaker (120 rpm). For the darkness condition, the 12-well plate containing films and controls were kept in the dark for 5 min and incubated under the same aforementioned conditions.

Bacterial cell survival for both irradiated and dark conditions was evaluated by counting colony-forming units (CFU) on MHA, as was previously described [35]. Briefly, aliquots of suspensions (75 µL aliquots of samples and controls) were serially diluted 10-fold in sterile distilled water to give concentrations of 10^−1^ to 10^−5^ times. Drops (5 µL) of each dilution and the original suspension were spotted onto MHA plates and incubated at 35 °C for 24 h. Each experiment was performed in duplicate on three independent occasions.

*Catalase assay:* it was carried out in 12-well sterile plate with two replicas of each polymer condition (PHEMA, PHEMA(Cl), PHEMA (I), RB@PHEMA(Cl) or RB@PHEMA(I)) and two with sterile water. All 12-well plates contained 800 µL of sterilized water together with the corresponding polymer. Then, the sterile plate was irradiated by white light for 1 h (288 J/cm^2^ of total light dose) at room temperature and under agitation in an orbital shaker (120 rpm). Subsequently, polymers were removed and the 800 µL of supernatant, from each polymeric condition, were split into two 400 µL aliquots, one without catalase and the other one with 2 µL of 1/1000 catalase dilution (previously sterilized by filtration). Then, the aliquots were kept at room temperature for 1 h and under low agitation (100 rpm). Finally, 100 µL of the bacterial inoculum was added to each 400 µL aliquot to obtain a final microbial suspension of ≈10^8^ bacteria/mL and were kept at room temperature for 5 min and under agitation (120 rpm). Bacterial cell survival for both with/without catalase conditions was evaluated by counting colony-forming units (CFU) on MHA, following the same experimental procedure described above. Each experiment was performed in duplicate on three independent occasions.

### 4.7. Statistical Analysis

The aPDI experiments (including dark conditions) were performed in three independent replicates for each bacterial species. Results are expressed as mean ± standard deviation. Mean comparisons were performed after verifying the homogeneity of variances using Barlett’s test. In cases of homoscedasticity, differences among treatments were analyzed by one-way analysis of variance (ANOVA) followed by Tukey’s multiple comparison test. If heterogeneity of variances existed, robust tests were carried out as follows. Welch’s test was used to check for differences among treatments, with Game-Howells’s test used to establish differences among treatments. Comparisons of the means with p values less than or equal to 0.01 were regarded as significantly different in all tests. All statistical analyses were performed using the IBM SPSS Statistics, version 27 (SPSS Inc., Chicago, IL, USA).

## Figures and Tables

**Table 1:** Formulations (Weight Parts) Used to Obtain PHEMA Films Described in this Study.

Salt	Polymer	HEMA	PEGDMA	AIBN	RB	TBAI	TBACl	NaI
No salt	PHEMA	85	15	1	0	0	0	0
	RB@PHEMA	85	15	1	0.1	0	0	0
TBAI	PHEMA(I)	85	15	1	0	10	0	0
	RB@PHEMA(I)	85	15	1	0.1	10	0	0
	RB@PHEMA(I)b	85	15	1	0.1	5	0	0
	RB@PHEMA(I)c	85	15	1	0.1	2.5	0	0
	RB@PHEMA(I)d	85	15	1	0.1	1	0	0
	RB@PHEMA(NaI)	85	15	1	0.1	0	0	10
TBACl	PHEMA(Cl)	85	15	1	0	0	10	0
	RB@PHEMA(Cl)	85	15	1	0.1	0	10	0

**Table 2:** TGA Data of PHEMA Films.

Polymer	T_5%_ (°C)	T_20%_ (°C)	T_max_ (°C)
RB@PHEMA(I)	258.7	303.8	333.7
PHEMA(I)	240.7	297.4	323.6
RB@PHEMA(Cl)	255.9	293.4	315.4
PHEMA(Cl)	248.9	280.2	303.7
PHEMA	219.5	244.8	268.2
